# 2542. Attenuation of Polymyxin and Aminoglycoside-Associated Nephrotoxicity

**DOI:** 10.1093/ofid/ofad500.2159

**Published:** 2023-11-27

**Authors:** Cole Hudson, James E Smith, Brianna Eales, Xinli Liu, Shama Kajiji, Luan Truong, Vincent Tam

**Affiliations:** University of Houston College of Pharmacy, Houston, Texas; University of Houston College of Pharmacy, Houston, Texas; University of Houston, Pasadena, Texas; University of Houston College of Pharmacy, Houston, Texas; Emergent System Analytics, LLC, Clinton, Connecticut; Houston Methodist Hospital, Houston, Texas; University of Houston, Pasadena, Texas

## Abstract

**Background:**

The clinical use of aminoglycosides and polymyxins to treat multidrug-resistant Gram-negative bacterial infections is hindered by dose-limiting nephrotoxicity. Recently, there has been increased interest in reducing the nephrotoxic potential of these antibiotics to combat the antimicrobial resistance crisis. We identified zileuton, an FDA-approved anti-inflammatory drug, via a virtual screen indicating that it has desirable properties as a potential nephroprotectant. The objective of this project was to determine if zileuton can reduce nephrotoxicity associated with amikacin and polymyxin B in a rat model of acute kidney injury.

**Methods:**

Sprague-Dawley rats (n=10, both genders) were administered either amikacin (300 mg/kg) or polymyxin B (20 mg/kg) daily for 10 days. Doses were chosen based on prior pharmacokinetic studies to achieve a systemic exposure comparable to that in humans after a standard dose. Zileuton (4 and 10 mg/kg) was delivered intraperitoneally 15 minutes before antibiotic administration each day. Blood samples were collected at baseline and daily to determine serum creatinine concentration. Nephrotoxicity was defined as a ≥ 2x elevation of baseline serum creatinine. Time-to-event analysis and log rank test were used to compare the onset of nephrotoxicity in different cohorts. Histopathological analysis was conducted to characterize the extent of renal injury.

**Results:**

Animals receiving amikacin or polymyxin B alone had nephrotoxicity rates of 90% and 100% within 10 days, respectively. The overall rate was reduced to 30% in animals receiving adjuvant zileuton. The onset of nephrotoxicity associated with amikacin and polymyxin B was also significantly delayed by zileuton 4 mg/kg and 10 mg/kg, respectively. Additionally, histopathology confirmed reduced renal injury in animals receiving concomitant zileuton.

**Conclusion:**

This pilot study suggests that zileuton has the potential to attenuate nephrotoxicity associated with last-line antibiotics. This would allow these antibiotics to treat multidrug-resistant Gram-negative bacterial infections optimally without dose-limiting constraints. Further studies are warranted to optimize drug delivery and characterize pharmacokinetics to correlate with clinical dosing in humans.

**Disclosures:**

**All Authors**: No reported disclosures

